# Structural basis of Arpin homodimerization reveals cooperative inhibition of the Arp2/3 complex through dual-site engagement

**DOI:** 10.1093/procel/pwag021

**Published:** 2026-03-24

**Authors:** Liru Liu, Xuejiao Zhang, Yun Zhu, Mengchen Pu, Yuru Geng, Min Wang, Rongguang Zhang, Shen Ge, Sheng Ye

**Affiliations:** School of Biomedical Science and Medicine Engineering, Beihang University, Beijing 100191, China; Key Laboratory of Precision Diagnosis and Treatment Technology and Intelligent Imaging Equipment (Beihang University), Ministry of Industry and Information Technology, Beijing 100862, China; State Key Laboratory of Biomacromolecules, Institute of Biophysics, Chinese Academy of Sciences, Beijing 100101, China; State Key Laboratory of Biomacromolecules, Institute of Biophysics, Chinese Academy of Sciences, Beijing 100101, China; Protein Design Unit, dProtein Biotechnologies Co. Ltd, Beijing 102206, China; School of Biomedical Science and Medicine Engineering, Beihang University, Beijing 100191, China; Key Laboratory of Precision Diagnosis and Treatment Technology and Intelligent Imaging Equipment (Beihang University), Ministry of Industry and Information Technology, Beijing 100862, China; State Key Laboratory of Biomacromolecules, Institute of Biophysics, Chinese Academy of Sciences, Beijing 100101, China; State Key Laboratory of Biomacromolecules, Institute of Biophysics, Chinese Academy of Sciences, Beijing 100101, China; Protein Design Unit, dProtein Biotechnologies Co. Ltd, Beijing 102206, China; School of Biomedical Science and Medicine Engineering, Beihang University, Beijing 100191, China; Key Laboratory of Precision Diagnosis and Treatment Technology and Intelligent Imaging Equipment (Beihang University), Ministry of Industry and Information Technology, Beijing 100862, China; State Key Laboratory of Biomacromolecules, Institute of Biophysics, Chinese Academy of Sciences, Beijing 100101, China; School of Engineering Medicine, Beihang University, Beijing 100191, China

**Dear Editor,** 

Cell migration is a sequential and interrelated multi-step process that requires the dynamic reorganization of the actin cytoskeleton, wherein the actin-related protein (Arp) 2/3 complex remains the sole identified modulator of actin branching (Suraneni et al., 2012; Shaaban et al., 2020). Its activity is tightly controlled by nucleation-promoting factors (NPFs) and several endogenous inhibitors (Alekhina et al., 2017). Recently, Arpin, a lamellipodial inhibitor identified through its conserved acidic motif (Dang et al., 2013), has been reported to restrain cancer cell migration (Liu et al., 2016; Lomakina et al., 2016), yet its inhibitory mechanism remains debated. Although lacking the characteristic verprolin homology (“V”) and central (“C”) motifs of NPFs, Arpin still possesses the conserved C-terminal acidic (“A”) motif and has been demonstrated to be a competitive inhibitor of the Arp2/3 complex against VCA-domain-containing NPFs (Dang et al., 2013). The N-terminal region of Arpin exhibits striking sequence conservation across species, but its functional contribution to Arp2/3 binding remains poorly defined. This evolutionary conservation of the N-terminal region, when juxtaposed with its unidentified functional role, underscores the limitations arising from a lack of high-resolution structural data. Notably, previous biochemical and low-resolution structural studies have primarily focused on the flexible C-terminal acidic tail (CT) as the major Arp2/3-interacting element, leaving the structural organization and potential oligomerization of the N-terminal globular core largely uncharacterized. Available structural data provide only a low-resolution small-angle X-ray scattering (SAXS) envelope of full-length Arpin, depicting a tadpole-like shape: an ellipsoid-shaped N-terminal globular domain and an extended linear CT (Fetics et al., 2016). Moreover, conflicting cryo-EM studies have proposed distinct binding configurations of Arpin to the Arp2/3 complex, ranging from multiple potential CT-binding sites (Sokolova et al., 2017) to a single occupied site (Fregoso et al., 2022). These conflicting findings highlight the inherent complexity of the binding modes between Arpin and the Arp2/3 complex.

Despite Arpin’s overall tadpole-like shape, confirmed by the SAXS analysis (Fetics et al., 2016), the structural details of its globular core (the “tadpole head”) remain unresolved, hindering comprehensive elucidation of its inhibitory mechanism. To address this, we performed crystallographic studies on Arpin orthologs from diverse species (Fig. S1), including both full-length constructs and truncated variants (Fig. 1A). Ultimately, a crystal of zebrafish Arpin (residues 9–221) with a single-site mutation C133S (to avoid unnecessary inter-molecular interactions) diffracted to a highest resolution of 1.65 Å (see Supporting Information for details; Fig. 1B, Table S1). Within one asymmetric unit, two Arpin molecules form a homodimer exhibiting pseudo two-fold rotational symmetry (Fig. S2A). Notably, residues 187–221 at the C-terminus are untraceable in the final models of both protomers, a feature consistent with their intrinsic flexibility and the tadpole-like conformation derived from the SAXS envelope. Accordingly, we define the globular core of Arpin as residues 1–186, while the CT of Arpin as residues 187–221.

**Figure 1.**
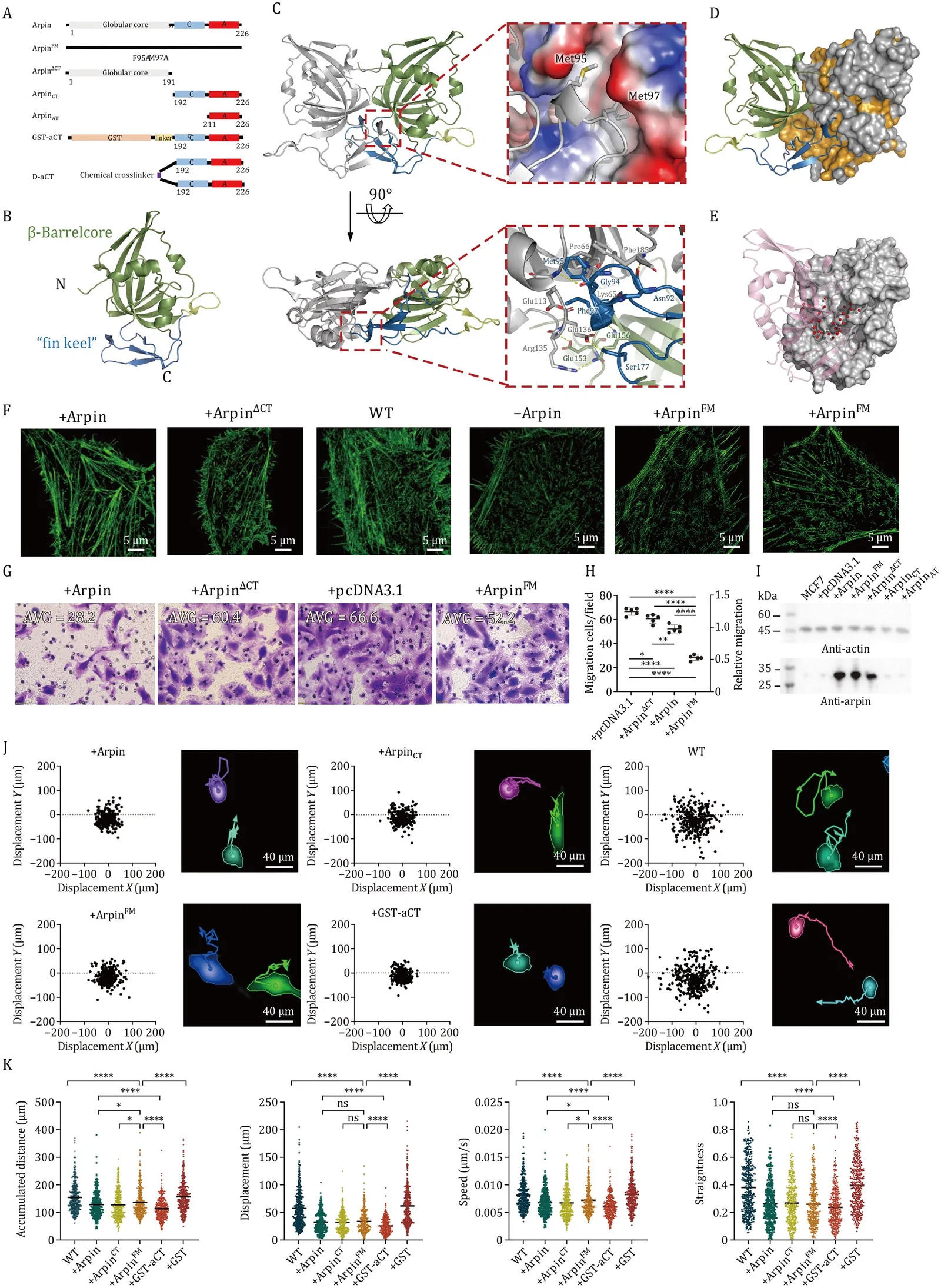
**Arpin homodimerization underlies its structural organization, biochemical activity, and suppression of Arp2/3-dependent directional migration**. (A) Schematic representation of truncated and mutated Arpin variants. The globular N-terminal domain is shown in grey, the central motif in blue, and the acidic tail in red. (B) Cartoon representation of the globular core of Arpin with β-barrel core (green), “fin keel” (blue) and a short linker (yellow) properly labeled. (C) Close-up view of the homodimerization interface around contact cluster II. Upper panel: At the tip of the “fin keel”, the hydrophobic side chains of Phe95 and Met97 are shown in sticks. Lower panel: Residues involved in the interactions between adjacent “fin keel” structures are shown in sticks, with inter-protomer hydrogen bonds indicated by dashed lines. (D) Conserved surface patch of Arpin. One protomer of the Arpin homodimer is shown in ribbons, while the other one as surface representation colored by sequence conservation (strictly conserved residues in orange, while others in gray). (E) Water-filled cavity at the Arpin homodimerization interface. A fully enclosed cavity is formed housing 35 ordered water molecules (in red balls) stabilized by a hydrogen-bond network. (F) Cytoskeletal staining of MCF-7 cells overexpressing Arpin or its variants. Confocal images of FITC labeled phalloidin-stained MCF7 cells showing the state and distribution of actin filaments (Scale bars, 5 nm). Cells were overexpressed with Arpin, Arpin^ΔCT^ and Arpin^FM^, respectively, as well as knocked down Arpin (-Arpin) and WT cell line. (G) Transwell assays were performed to analyze the effects of transfection of Arpin with its variants on the migration of MCF-7 cells. Representative images of crystal violet staining of cells captured 24 h after treatment, original magnification 40×. (H) Numbers of migrated cells through the chamber after 24 h. Cells were counted from 10 random microscope fields for each sample in 3 independent experiments. Statistical significance is indicated as follows: *P < 0.05; **P < 0.01; ***P < 0.001; ****P ≤ 0.0001; ns, not significant (*P *> 0.05). (I) Western blot confirms the expression levels different across Arpin constructs. (J) Representative migration trajectories and vector displacement analysis of the time-lapse tracking of HT1080 cells with stable Arpin expression. HT1080 cells with stable expression of Arpin or control were seeded on collagen-coated dishes and imaged for 6 h. Each cell’s starting position was set as the origin, and its final position defined the endpoint for vector displacement analysis. Representative migration tracks were also selected and shown to illustrate typical movement patterns. (K) Accumulated distance, net displacement, migration speed, and directionality (straightness) of migrating cells were measured in three independent experiments. Data are presented as mean ± standard deviation. Statistical significance is indicated as follows: *P < 0.05; **P < 0.01; ***P < 0.001; ****P ≤ 0.0001; ns, not significant (*P *> 0.05).

The overall structure of Arpin’s globular core indeed represents an ellipsoid-shaped domain, characterized by an antiparallel β-barrel framework containing six β-strands (Figs. 1B and S2B). Sequence alignments show remarkably low homologies of Arpin with previously known structures (< 20% similarity), yet its architecture adopts the canonical oligonucleotide/oligosaccharide-binding fold (OB-fold) (Fig. S3A). Structural analysis with the DALI server (Holm and Laakso, 2016) revealed that Arpin shares the highest structural similarity with two distinct classes of OB-fold proteins (z-score of 6.4): single-stranded DNA-binding proteins (SSBs) from bacteria or yeast mitochondria and metallochaperone proteins in bacterial cation efflux systems (Fig. S3B and S3C). Despite the obvious structural parallels, Arpin displays notable functional differences from either SSBs or metallochaperones.

The most surprising structural finding of Arpin is its face-to-face homodimeric configuration within a single asymmetric unit, whereby two Arpin protomers assemble into a homodimer with a buried interface area of ∼1,722 Å^2^ (16% of the total solvent-accessible surface area). PISA (Krissinel and Henrick, 2007) analysis indicated that this interface confers high complex formation significance, whereas each of the other contacts observed in the crystal buries a substantially smaller interface area (approximately 187 Å^2^ and 50 Å^2^) and corresponds to non-specific crystal packing interactions. The two protomers packed with each other in a two-fold rotational symmetric manner, with their β-barrels approximately parallel to each other, resembling the shape of a butterfly (Fig. 1C). Interestingly, the entire “fin keel” region of the “butterfly” is the most conserved moiety of Arpin (Figs. 1D and S5), including an absolutely conserved motif ranging residues 90–105 (β6, β7, η3, and connecting loops). Notably, these conserved residues reside within the N-terminal globular core; this region remained unresolved in previous low-resolution SAXS and cryo-EM studies and, as a consequence, was not investigated in earlier biochemical analyses. The strict conservation of the “fin keel” structure strongly implies that both inter-protomer interactions and the resulting homodimeric configuration are conserved across various species.

Approximately 20% of residues within the Arpin globular core participate in the homodimerization interface and form an extensive network of intermolecular interactions, which can be further divided into two distinct contact clusters (Figs. 1C and S4). Notably, a substantial enclosed cavity lies between the two clusters (1,628 Å^3^, calculated using MOLE 2.0; Fig. 1E) and is filled with 35 ordered water molecules (average *B*-factor of 40.0 Å^2^; Table S1), which are only partially visible in the experimentally phased low-quality electron density map (Fig. S6). When the Arpin homodimer is properly formed, its central cavity becomes inaccessible to solvent, preventing water exchange between the cavity and the solvent. The tightly restricted mobility of these water molecules further enables their observation in the crystal structure. Importantly, we do not imply a specific functional role for these ordered water molecules. Instead, their ordered appearance likely reflects the restricted solvent accessibility and structural rigidity of the enclosed homodimer interface.

The final model of homodimeric Arpin structure encompasses only the globular core, yet the construct for crystallization actually includes the majority of the CT after the globular core. SDS-PAGE analysis of crystallized Arpin confirmed the CT to be intact, with no evidence of proteolytic degradation, suggesting that Arpin homodimerization is not an artifact caused by the putative loss of CT. Nevertheless, two unsolved critical questions persist: (i) whether Arpin exists as stable dimers under physiological conditions, and (ii) whether full-length human Arpin (hArpin) can also form homodimers as zebrafish Arpin does.

To address these questions, size-exclusion chromatography (SEC) and analytical ultracentrifugation (AUC) analyses were carried out; and the results showed that recombinantly expressed hArpin and zebrafish Arpin both form homodimers in solution (Fig. S7A and S7B). Notably, SDS–PAGE analysis of fractions corresponding to the apparent monomeric and dimeric peaks revealed the coexistence of both species, indicating that Arpin undergoes reversible monomer–dimer interconversion in solution. Therefore, in this study, the terms “dimeric Arpin” and “monomeric Arpin” refer to dimer-enriched and monomer-enriched populations in *in vitro* assays, respectively, rather than strictly isolated or constitutively stable oligomeric states. In addition, our results demonstrate that zebrafish Arpin forms homodimers preferentially at high concentrations (e.g., in concentrated samples or tightly packed crystalline states), whereas hArpin readily undergoes homodimerization even at substantially lower concentrations.

Structural analysis of the Arpin homodimer further revealed that two highly conserved hydrophobic residues, Phe95 and Met97, which localize to the so-called fin keel moiety, are critical for the integrity of the homodimerization interface. SEC and AUC analyses demonstrated that the F95A/M97A mutant (designated as hArpin^FM^) drastically impairs the stability of the Arpin homodimer (Fig. S7C and S7D). Given that this double-site mutation exclusively abrogates the hydrophobic interactions between the fin keel moieties of the two protomers without directly affecting the extensive intermolecular hydrogen-bond network mediated by ordered water molecules at the interface, these findings highlight that the hydrophobic interactions mediated by Phe95, Met97 and related hydrophobic residues are the key determinant for the homodimerization of Arpin, while the water-bridged hydrogen-bond network confers sufficient flexibility and dynamics to the homodimeric state of Arpin.

Previous studies have suggested that the Arp2/3 complex contains two distinct binding sites for Arpin, implying the possibility of multivalent interactions (Sokolova et al., 2017). Our structural and biophysical data reveal that Arpin forms homodimers in solution, raising the possibility of a 2:1 Arpin: Arp2/3 complex assembly that could cooperatively block both CT-binding sites. In the context of this work, cooperative inhibition describes a homodimerization-dependent mode of Arp2/3 complex inhibition, wherein dual-site binding confers enhanced inhibitory potency relative to monomeric Arpin. To evaluate the functional relevance of this mechanism, we examined cytoskeletal organization using breast cancer cell line MCF-7 as a model system, which expresses minimal endogenous Arpin (Lomakina et al., 2016; Montoya-Garcia et al., 2024). Unless otherwise specified, all subsequent experiments were performed using human Arpin. Cytoskeletal staining showed that Arpin (referring to hArpin in this and subsequent cell assays) overexpression produced thicker and longer F-actin filaments, whereas Arpin knockdown resulted in thinner, less extensive actin bundles, consistent with its role in suppressing branched nucleation (Dang et al., 2013; Montoya-Garcia et al., 2024) (Fig. 1F). The CT of Arpin contains an acidic tail region that is essential for Arp2/3 inhibition. To dissect the functional contribution of this region, we generated a series of constructs that either remove or isolate it (Fig. 1A), including Arpin^ΔCT^ (residues 1–191, lacking the entire acidic tail region), Arpin_CT_ (residues 192–226) and Arpin_AT_ (residues 211–226). Arpin_AT_ comprises only the minimal acidic “A” motif required for Arp2/3 inhibition. In contrast, Arpin_CT_ encompasses a longer acidic tail that includes both the “A” motif and its upstream flanking region. As expected, the variant Arpin^ΔCT^ failed to reproduce the branching inhibitory effects of full-length Arpin, confirming the essential role of the acidic tail (Fig. 1F). Strikingly, MCF-7 cells expressing Arpin^FM^ exhibited a dendritic microfilament structure lacking long bundles, mimicking the effect caused by Arpin^ΔCT^ rather than full-length Arpin (Fig. 1F). Conclusively, Arpin’s ability to inhibit microfilament branching can be impaired by the double-site mutation F95A/M97A.

Given that Arpin^FM^ retains an intact C-terminal acidic tail, we next asked whether disruption of homodimerization affects its interaction with the Arp2/3 complex. Surface plasmon resonance (SPR) assays directly comparing Arpin variants revealed that Arpin homodimerization synergistically enhances its binding to Arp2/3 (Fig. S8; Table S2). Notably, Arpin^FM^ exhibited even weaker activity than monomeric-enriched Arpin, indicating that the F95A/M97A double-site mutation disrupts the cooperative binding by preventing homodimerization.

Having established the functional interplay between Arpin’s homodimerization and its Arp2/3-binding activity, we proceeded to investigate how this cooperative mechanism influences cell migration. Therefore, we implemented multiple approaches to examine both directional migration capabilities and real-time migratory dynamics in cancer cells expressing Arpin or its engineered variants, including Arpin^FM^, Arpin^ΔCT^, Arpin_CT_, and Arpin_AT_ (Fig. 1A). Transwell assays performed in MCF-7 cells (Fig. 1G, 1H, and 1I) showed that the F95A/M97A mutation caused a pronounced loss of inhibitory activity, clearly demonstrating that homodimerization is essential for the robust ability of Arpin to suppress cell migration. We further evaluated the dynamic migratory behavior of HT1080 fibrosarcoma cells (Fig. 1J; Movie S1). The reduction in net displacement, with no remarkable decrease in accumulated distance, indicated that Arpin does not significantly reduce migration speed but instead decreases directional persistence, consistent with previous reports (Dang et al., 2013). Moreover, among the three Arpin constructs evaluated, full-length Arpin and Arpin_CT_ exhibited similar inhibitory capacity (*P *> 0.1; Fig. 1K), whereas Arpin^FM^ showed weaker inhibitory functions than the other two constructs (*P *< 0.1; Fig. 1K). In other words, fusing a monomeric N-terminal domain to the CT even attenuated the CT’s inhibitory function. These results suggest that the evolutionary significance of Arpin’s N-terminal globular domain is inextricably coupled with its homodimerization ability.

To create an Arp2/3 inhibitor with greater potency than Arpin and further validate the 2:1 binding model, we sought to identify a naturally occurring cytosolic protein to replace the N-terminal globular domain of Arpin. Ultimately, glutathione S-transferase (GST) was selected due to the following reasons: (i) it has been structurally and biophysically characterized as a homodimer with enhanced stability relative to Arpin; (ii) its homodimeric architecture displays two-fold rotational symmetry; and (iii) two C-terminal extensions of its homodimer align in the same spatial orientation with inter-terminal distances comparable to those observed in Arpin homodimers. Then, we designed a chimeric protein with Arpin’s CT fused to the C-terminus of GST (designated as GST-aCT, Fig. 1A). Structural comparison of Arpin and GST homodimers revealed inter-terminal Cα distances of 25.0 Å (Fig. S10A) and 46.7 Å (Fig. S10B), respectively. Simulations using AlphaFold 3 (Abramson et al., 2024) indicated that a short 4 residue spacer (-GSSG-, Table S2) allows the two CTs of GST-aCT homodimer access to both Arp2/3 binding sites (Fig. 2A). Recombinant GST-aCT formed stable homodimers on SEC and showed markedly enhanced Arp2/3 affinity in SPR assays (Fig. S8, Table S2). Consistently, live-cell imaging of HT1080 cells expressing GST-aCT revealed stronger suppression of directional migration than full-length Arpin or other variants (Fig. 1J and 1K). These results suggest that the N-terminal domain facilitates homodimerization and stabilizes a higher-order Arpin–Arp2/3 assembly adopting a 2:1 binding model, which can be further enhanced by replacing the N-terminal globular core of Arpin with more stable homodimerization proteins.

**Figure 2.**
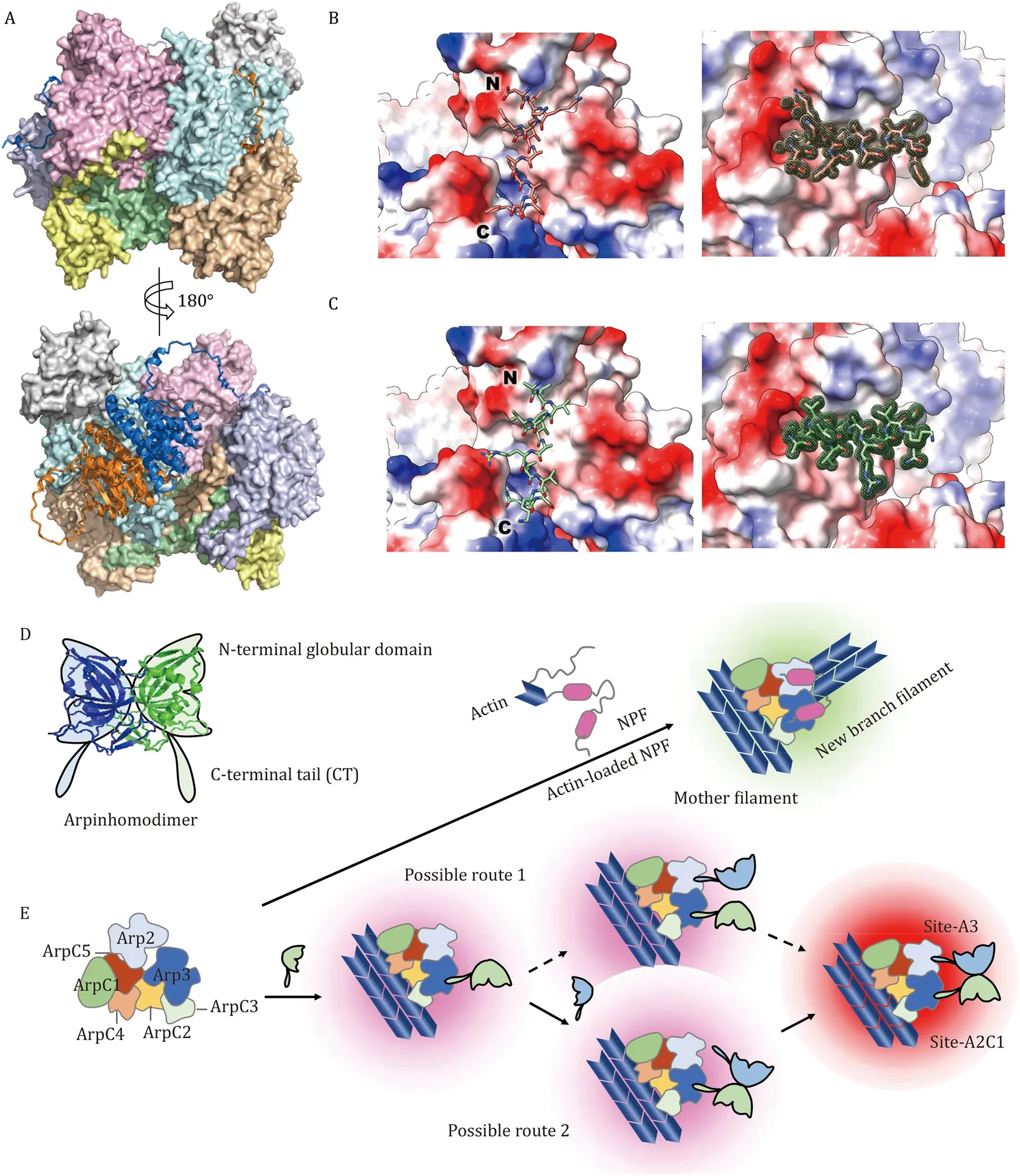
**AI-engineered dual-tailed peptides and the proposed “butterfly” model of Arpin-driven cooperative inhibition of the Arp2/3 complex**. (A) Structural modeling of GST-aCT bound to the Arp2/3 complex using AlphaFold 3. Simulated 2:1 binding stoichiometry confirms that a short linker with 4 residues enable spatial accessibility of both Arp2/3 binding sites. (B) Detailed binding pose of the AI-designed dual-tailed peptide dP_arpin_0548 and its CUTEDGE-generated electron densities. (C) Detailed binding pose of the AI-designed dual-tailed peptide dP_arpin_1071 and its CUTEDGE-generated electron densities. (D) The shape of Arpin’s homodimeric conformation resembling a butterfly. The two globular domains form the “wings,” while the unobserved flexible CT are conceptualized as “cerci.” (E) Schematic model of two potential inhibitory pathways. Upper panel: In the “green light” mode, Arp2/3 binds NPF and actin to promote microfilament branching. Lower panel: In the “red light” mode, Arpin competitively occupies the Arp2/3 binding sites through two possible pathways: (1) sequential binding at site-A3 and site-A2C1 followed by dimerization, or (2) initial homodimerization, followed by cooperative dual-site engagement.

Extending this strategy, we designed a minimal dual-tailed peptide (D-aCT) comprising two Arpin CTs linked at their N-termini via a 16-residue spacer (sequence: -(SSGG)_4_-) and a cysteine-mediated BM(PEG)_3_ crosslinker (Figs. 1A and S11). Both SEC analysis and mass spectrometry (MS) confirmed that D-aCT formed stable covalent dimers via BM(PEG)_3_, rather than through reducible disulfide bonds. Subsequent SPR assays revealed that D-aCT bind to Arp2/3 with a *K*_D_ of 5.5 μmol/L (Fig. S12; Table S2). This affinity is comparable to that of GST-aCT (2.5 μmol/L) and surpasses that of full-length Arpin or a single Arpin_CT_ peptide (14.1 and 17.8 μmol/L, respectively), demonstrating that the much smaller D-aCT achieves robust binding activity comparable to that of larger Arpin-based constructs.

To further enhance D-aCT’s binding affinity for Arp2/3, we employed CUTEDGE (Chain-free Underinformed Targeted Electron Density Generative Explorer), a 3D diffusion-based deep generative framework for peptide binder design. Previous studies have identified that Arpin engages the Arp2/3 complex through two distinct CT-binding sites: one on the Arp3 subunit (designated “site-A3”) (Fregoso et al., 2022) and the other being a putative site located within the cleft formed by subunits Arp2 and ArpC1 (designated “site-A2C1”) (Saks et al., 2025; Zimmet et al., 2020), providing the rationale for designing dual-interface peptides. Using the cryo-EM structure of Arpin_CT_: Arp2/3 (PDB entry: 7JPN) as the target model for designing, CUTEDGE generated 600 electron density maps of 13-residue α-helical peptides to replace the central helical region of Arpin_CT_, which were ultimately refined into 2,292 sets of structural coordinates. Computational evaluation across both binding sites (Fig. 2B and 2C) included a rigid-body docking simulations (Fig. S13) and a subsequent multi-parameter scoring pipeline (partial sequences with high scores are listed in Tables S3 and S4). This framework identified six structurally viable peptides with predicted binding free energy comparable to that of native Arpin. These sequences were introduced into D-aCT scaffolds properly (Table S2) and named after the ID number of the designed peptides. Among them, two designed dual-tailed peptides, dP_arpin_0548 (*K*_D_ = 0.70 μmol/L) and dP_arpin_1071 (*K*_D_ = 5.51 μmol/L), showed further enhanced affinity in SPR assays (Fig. S12). In fact, dual-tailed dP_arpin_0548 exhibited a 20-fold improvement in binding affinity compared to homodimeric Arpin and a 7.9-fold increase over D-aCT, demonstrating that scaffold-constrained design via CUTEDGE can achieve dual-interface compatibility and a further enhanced binding affinity of the Arp2/3 complex.

These findings underscore that dimerization is indispensable for the suppressive activity of Arpin, suggesting that its cooperative assembly may serve as a critical node in the regulation of metastatic progression. Based on our findings, we propose the “butterfly” model to elucidate the assembly logic of Arpin-Arp2/3 interactions. The crystal structure of the Arpin homodimer reveals two protomers resembling the “wings” of a butterfly, with their missing CT segments extending outward as flexible “cerci” (Fig. 2D). The model centers on a two-step binding process. First, initial preferential occupation of site-A3 by the CT of a single Arpin molecule leads to the formation of a stable 1:1 complex, which inhibits the short-pitch transition of NPF binding by locking the inhibitory tail of Arp3 at the high-affinity site-A2C1 (Shaaban et al., 2020; Zimmet et al., 2020), and competes with NPF for the low-affinity binding site on Arp3 (Fregoso et al., 2022). Second, a subsequent binding pathway of the second Arpin molecule could display dynamic preference (Fig. 2E). One possibility involves directly engaging Arpin’s CT with site-A2C1, albeit with weaker affinity. Spatial effects then promote proximity between the two Arpin molecules, facilitating homodimerization—two “wings” assembling into a complete “butterfly”. This dimerization stabilizes the complex via interactions at the unstable homodimerization interface and low-affinity site-A2C1, collectively forming a stable oligomer with three distinct interaction sites. However, experimental evidence strongly supports a more efficient pathway: the second Arpin molecule first associates with the pre-bound Arpin to form a homodimer. This intact “butterfly” spatially reorients the second “cercus” toward site-A2C1, significantly boosting CT-binding efficiency at site-A2C1 and stabilizing a structurally robust oligomer. Notably, as a more stable homodimer, engineered GST-aCT recapitulates this pathway, exhibiting superior Arp2/3 binding compared to monomeric Arpin and enhanced inhibition of cellular motility.

Regarding the positive cooperativity proposed in the butterfly model, further experimental validation is required. One potential approach involves employing *in situ* EM methods for structural characterization of Arpin: Arp2/3 complexes bound to actin filaments. Nonetheless, the low molecular weight of Arpin poses significant technical challenges for such studies. Alternatively, stabilized GST-aCT homodimers could be directly utilized for structural analysis of the complex. This methodology is precedented by studies of the GST-Crn1: Arp2/3 complex, which successfully resolved its 2:1 binding configuration (Sokolova et al., 2017). Another complementary strategy entails preparing stabilized Arpin homodimers via chemical crosslinking, followed by structural characterization of the resultant 2:1 Arpin: Arp2/3 complexes. Additionally, experimental investigation of the asymmetry in positive cooperativity requires site-directed mutagenesis of the two binding sites on Arp2/3. This could be achieved through knockout/knock-in strategies to generate Arp2/3 complexes with mutations at individual binding sites, enabling verification of the dynamic binding order between the two sites and the determination of independent binding affinity measurements unaffected by cooperative effects. Collectively, these approaches aim to dissect the molecular basis of cooperative binding and validate the mechanisms proposed in the butterfly model.

This study reveals a critical interplay between Arpin homodimerization and its inhibitory function in regulating cell migration, and demonstrates a pronounced positive cooperative effect mediated by homodimerization. However, two key questions remain to be addressed. First, why does Arpin form dynamic homodimers instead of stable ones, and what is the significance of this dynamic behavior in regulating its inhibitory activity? A potential regulatory mechanism may involve as-yet-unidentified factors that modulate Arpin’s activity through disruption of its homodimerization, rather than through direct competition for the AT-binding site. Second, what underlies Arpin’s intriguing inhibitory mechanisms, which minimally reduce migration velocity yet paradoxically enhance steering capacity? Steering depends on *de novo* lamellipodia formation via actin branching networks, whereas directional persistence relies on the stabilization of existing networks. Our data indicate that Arpin’s function does not directly inhibit the formation of new branches but instead facilitates the disassembly of pre-existing networks or prevents rebranching during their turnover. Further investigation into the molecular mechanism of Arpin’s homodimerization-dependent inhibition, coupled with its dynamic regulation, may pave the way for developing Arpin-based therapeutic strategies to combat cancer metastasis.

## Supplementary Material

pwag021_Supplementary_Data

## Footnotes

We are very grateful to the staff of the Structural Biology Core facility (Institute of Biophysics, Chinese Academy of Sciences) for their technical assistance, especially to Ms. Ya Wang, Ms. Xiaoxia Yu, Ms. Yuanyuan Chen and Mr. Yi Han. We would like to thank Ms. Shuoguo Li and Miss. Yun Feng from the Center for Biological Imaging (CBI, Institute of Biophysics, Chinese Academy of Science) for their help of taking and analyzing SIM images. We would also like to thank the Biological Reaction Facility (Ms. Wenjuan Zhang) and the Imaging Facility (Mr. Jun Chen and Miss. Chunhua Zhang) of the National Center for Protein Sciences Beijing for their assistance.

This work was supported by the National Science and Technology Major Project of China (Grant No. 2026ZD1218801), the National Natural Science Foundation of China (Project No. 31871287, 31400638 and 31470792), and Beijing Municipal Science & Technology Commission grant (Z221100003522020).

The authors declare no competing interests. Coordinates and structure factors have been deposited in the Protein Data Bank with accession number 25YR for crystal structure of zebrafish Arpin.

L.L. and X.Z. performed most of the biochemical experiments; Y.Z., R.Z. and S.Y. carried out the crystal data processing and structure refinement; M.W. and Y.G. participated in the SPR experiments; M.P. and S.G. provided the AI algorithms and analysis tool, and participated in the interpretation of the data; L.L., M.P. and S.Y. prepared the related figures; S.Y. designed and supervised the project; L.L., X.Z., Y.Z. and S.Y. wrote the manuscript. All authors have given approval to the final version of the manuscript. No AI-assisted technologies were used in the preparation of this manuscript.
