# Supplementary material for: Structural basis of Arpin homodimerization reveals cooperative inhibition of the Arp2/3 complex through dual-site engagement
Source: Protein Cell. 2026 Mar 24;17(10):967–74. doi: 10.1093/procel/pwag021 (PMC13626732; doi:10.1093/procel/pwag021)
Supplement: Supplementary file 1 [file pwag021_supplementary_data.zip › Arpin_Protein Cell_Supplemental Files_Revised_Liu_et_al_260520/Liu et al.,_Supporting Information_Revised.docx]

**Supporting Information for**

Structural basis of Arpin homodimerization reveals cooperative inhibition of the Arp2/3 complex through dual-site engagement

Liru Liu, Xuejiao Zhang, Yun Zhu, Mengchen Pu, Yuru Geng, Min Wang, Rongguang Zhang, Shen Ge & Sheng Ye

Correspondence to: Sheng Ye, Professor, Ph.D.

Email: yesheng@buaa.edu.cn (S. Ye)

**This PDF file includes:**

Supporting materials and methods

Tables S1 to S4

Figures S1 to S13

Movies Legends for S1

## Protein expression and purification

The human and zebrafish Arpin genes (*Homo sapiens*, NM_182616, *Danio rerio*, NM_001017780, including truncations and mutants) were synthesized (Sangon Biotech, China) and inserted into expression vector pET-32a. The fusion protein was overexpressed in *Escherichia coli* BL21. The F95A and M97A mutation was introduced by double mutant site-direct mutagenesis and verified by sequencing. Cells were grown to OD600≈0.8 in LB media supplemented with 1 µg/ml ampicillin at 37℃ and were induced by 1 mM IPTG for 16 hours at 4℃ for expression. Cells were harvested by centrifugation and lysed by high pressure homogenizer for twice after resuspended in buffer containing 50mM Tris-HCl pH 8.0, 200mM NaCl. The fusion protein was isolated from the soluble cell lysate by Ni-affinity chromatography. After using PreScission Protease to cleaves the expression tag, Arpin was purified by Hitrap Q HP column (GE Healthcare Biosciences, USA) for anion-exchange chromatography and a Superdex-200 gel filtration column (GE Healthcare Biosciences, USA). The purified protein was concentrated to 25mg/ml for crystallization or storage at -80℃.

## Crystallographic studies on Arpin

Both N- and C-terminal truncations with various lengths were screened to avoid the interference in the crystallization process, and a construct containing residues 9-221 eventually resulted in crystals, which, however, had a poor x-ray diffraction capability and could not be further optimized. Therefore, we introduced a single site-directed mutation of C133S to avoid unnecessary surface interactions. 1μl of protein solution (10 mg/ml in buffer of 50mM Tris-HCl pH 8.0, 200mM NaCl) and 1μl of reservoir solution (1.6 M Sodium citrate pH6.5) were mixed. The droplets were set on a siliconized glass sheet and inverted on the chamber with 1ml of reservoir solution and then sealed with vacuum silicone grease. Crystals formed after four days. Then, we obtained Arpin crystals in the space group of P6_5_ that diffracted to a highest resolution of 1.65 Å. To solve the phasing problem, Arpin was first subjected to a Blastp run against the Protein Data Bank (PDB) to find a search model for the molecular replacement method. Nevertheless, no structures with high similarities could be found, except the reported C-terminal tail structure of *hs*Arpin (PDB entry: 4Z68). Then, we soaked Arpin crystals in heavy-atom solutions and successfully introduced mercury ions into the crystals. Eventually, a single-wavelength dispersion dataset of a Hg derivative Arpin crystal was collected and processed to 2.1 Å. The anomalous scattering signals were strong enough to locate the Hg sites but insufficient to solve the phases. Therefore, the native dataset was combined with the Hg derivative one to perform single isomorphous replacement phasing, which successfully led to proper initial phases and a subsequent initial model. The model of Arpin was subsequently refined against the 1.65-Å native dataset to a final *R*_work_/*R*_free_ of 0.169/0.194. The native data sets and heavy atom derivative data sets were all collected at beamline BL-18U1 of Shanghai Synchrotron Radiation Facility with wavelength of 0.9778 Å for the Arpin crystals. The crystals were kept at 100 K during X-ray diffraction data collection. Data were indexed and scaled with HKL2000. Phases were solved by single-wavelength anomalous dispersion method using PHENIX. autosol. All refinement procedures were carried out with PHENIX. refine and COOT. Table S1 shows the detailed statistics of data collection and refinement.

## Multiple sequence alignment of Arpin

According to the multiple sequence alignment of Arpins from different species, there are three strictly conserved motifs (referred to as Motif 1 to 3, respectively). Both Motif 1 and 2 belong to a segment between β-strands β3 and β8 (residues 72-137) that actually includes most of the appendages to the β-barrel (helices α2, α3 and η2 and β-strands β4, β5, β6 and β7). Motif 1 (^77^KGNEIEPNFS^86^) is implicated in the formation of a β-hairpin β4-β5, which is the most flexible region of Arpin and adopts different conformations in both protomers. Motif 2 (^90^KVNTGFLMSSYKVEAKG^106^) contains β6, η2 and β7, which form a hairpin structure folded back to the surface of the central β-barrel. The two β-strands, β6 and β7, further compose a 3-stranded β-sheet with the C-terminal β-strand β11 (β6/7/11 hereafter), such that the C-terminus is tightly clamped between the β-barrel and the folded hairpin structure. In addition, a disulfide bond is formed between Cys110 and Cys183, which might not exist in cytosolic Arpins and is not conserved among species. It is interesting to note that β11 is located in Motif 3 (^178^GTVTKCNF^185^), making β-sheet β6/7/11 the most conserved region of Arpin. In fact, β6/7/11 are deeply involved in the homodimerization of Arpin by forming the “fin keel” structure, indicating an important role for Motif 2 and 3.

## Structural conservation of Arpin

Based on the structure of Arpin, we succeeded to identify a number of unique features, including two extra regions as appendix to the central OB-fold, and the previously unknown dimeric state of Arpin with a novel homodimerization pattern. These facts urged us to find out whether the structural features we observed can be shared by Arpins from human and other species.

To assess the structural conservation of Arpin, we submitted the structure of Arpin to ConSurf server. Searching against a non-redundant protein sequence database led to 143 unique hits, including Arpins from different species, as well as predicted Arpins. Based on multiple sequence alignment of the hits, conservation scores of each residue were subsequently calculated via Bayesin method^1^, resulting in values ranging from -1.2 to 2.9, where negative and positive values represent conserved and variable residues, respectively. Since the scores provided by ConSurf have been normalized with an average value of 0 and a standard deviation of 1, the result range indicates that the sequence of Arpin have more conserved residues than variable ones.

As expected, the β-barrel core of OB-fold is relatively conserved. At the homodimeric interface, contact clusters I and II exhibit distinguished conservation degrees. Residues around contact cluster I, as well as the residues constituting the hydrophilic groove below it, are mostly variable among species, even harboring the highest conservation score in Arpin residues (2.9 for Arg135). In contrast, residues around contact cluster II are generally conserved, especially around the hydrophobic pocket and the protruding extra β-sheet β6/7/11, indicating that the extensive interprotomer interactions around cluster II are also conserved among species. Since, comparing to cluster I, contact cluster II contributes more to the formation of Arpin homodimers, we reasoned that the homodimeric form of Arpin is highly possible to be shared by Arpins from human and other species.

## Surface plasmon resonance

All SPR experiments were performed using Biacore 8K instruments (GE Healthcare) at 25 °C, with PBS containing 0.05% (v/v) Tween-20 as the running buffer. Ligands were immobilized on CM5 sensor chips via standard amine coupling, following the manufacturer’s instructions (GE Healthcare). Kinetic parameters were derived by fitting the sensorgrams to fit binding model. Increasing concentrations of Arpin variants were injected at a flow rate of 30 μL/min, with a 60s association phase followed by a 60s dissociation phase. For Arp2/3 complex binding assays, dimeric and monomeric fractoins of Arpin, as well as Arpin^FM^, were tested in a concentration range of 3.125–200 μM, while Arpin_CT_, GST-aCT and D-aCT were tested at 0.625–40 μM. AI-designed Arpin variants were tested in a concentration range of 0.156–10 μM. Capture levels were optimized to achieve appropriate analyte responses. The anti-tag capture antibody was covalently coupled to the CM5 chip via amine coupling and regenerated according to the manufacturer’s protocol.

## Cell culture and transfection

The human breast cancer cell line MCF7 and fibrosarcoma cell line HT1080 were cultured in high-glucose DMEM without glutamine, supplemented with 10% FBS, at 37 °C, 5% CO₂. Mammalian codon-optimized synthetic human Arpin genes (including full-length, mutant, and truncated variants) were cloned into the pcDNA3.1 expression vector (Tsingke, China). Duplex siRNAs were synthesized as follows: Arpin knock down-specific sequence, 5′-GGCGCUAGUUGGACCGAUA-3′(sense) (Sangon Biotech, China).

Transient transfection of the human fibrosarcoma HT1080 cells was performed using lipofectamine 2000 (Invitrogen, USA) according to the manufacturer’s protocol. Briefly, 1 μg plasmid DNA encoding mScarlet-tagged Arpin variants (pcDNA3.1 vector) was diluted in 150 μL Opti-MEM to prepare the plasmid solution. 3 μL Lipofectamine 2000 was diluted in 150 μL Opti-MEM to prepare the liposome solution. Both mixtures were incubated at room temperature for 5 min. The plasmid solution was then added dropwise to the liposome solution and incubated for 20 min at room temperature. The resulting transfection mixture was added dropwise to 10 cm dishes containing HT1080 cells at approximately 80% confluency. Six hours post-transfection, the culture medium was replaced with high-glucose DMEM lacking glutamine and supplemented with 10% FBS. Protein expression was assessed 24 h later by flow cytometry.

HT1080 cells with stable mScarlet-tagged Arpin variants expression were also generated by lentiviral infection following the appropriate biological safety regulations. To deliver plasmids to generate the lentiviral particles encoding protein, lipofectamine 2000 was used according to the manufacturer´s protocol. 3.5μg of the plasmid pCDH-Arpin variants encoding the mScarlet protein was incubated with packaging plasmids (2.5 μg pLP1, 2.5 μg pLP2, 1.5 μg pLP-VSVG) in 200 μl Opti-MEM. The mixture was vortexed for 10 s. In a separate mix, 15 μl of lipofectamine 2000 was added to 200 μl Opti-MEM and was gently added to the first mixture followed by 5 min incubation at room temperature. The transfection mix was added drop-wise to a 10 cm dish of ~80% confluent HT1080 cells. 6 h post-transfection, the medium was replaced with high-glucose DMEM without glutamine, supplemented with 10% FBS. 48 h later, the lentiviral medium was collected and filtered through 0.45 µm filters. Target cells were spinfected with the optimized titer of the lentiviral medium by centrifugation at 4000 × rpm for 10 min at room temperature. Spinfected cells were recovered for one day in fresh medium and mScarlet expression was confirmed by flow cytometry. Lentiviral transduction was performed by adding viral particles to cells in culture medium at a 1:1 ratio (virus: DMEM) supplemented with polybrene (final concentration: 8 μg/mL). After 12 h incubation, the medium was replaced with fresh DMEM containing 10% FBS. Expression of the target gene with fluorescent tag was assessed 48 h infection. Stable cell lines were generated by subsequent puromycin selection. Cells were initially treated with 2 μg/mL puromycin, with the concentration gradually increased (up to 15 μg/mL) over several passages until a fully positive, stably expressing population was obtained.

## SIM Imaging of MCF7 Cells

MCF7 cells grown on glass coverslips were fixed with 4% paraformaldehyde in PBS for 10 minutes at room temperature, followed by three washes with PBS. The cells were then permeabilized with 0.5% Triton X-100 in PBS for 5 minutes. F-actin was stained using TRITC-conjugated phalloidin (Yeasen, China) by incubating the samples in the dark for 30 min. After additional PBS washes, the coverslips were mounted in glycerol mounting medium.

SIM images of MCF7 cells were acquired on the DeltaVision OMX V3 imaging system (GE Healthcare) with a ×100/1.40 NA oil objective (Olympus UPlanSApo), solid-state multimode lasers (488, 561nm) and electron-multiplying CCD (charge-coupled device) cameras (Evolve 512×512, Photometrics). Serial Z-stack sectioning was done at 125 nm intervals for SIM mode. To obtain optimal images, immersion oils with refractive indices of 1.516 were used for MCF7 cells on glass coverslips. The microscope is routinely calibrated with 100 nm fluorescent spheres to calculate both the lateral and axial limits of image resolution. SIM image stacks were reconstructed using softWoRx 6.1.1 (GE Healthcare) with the following settings: pixel size 39.5 nm; channel-specific optical transfer functions; Wiener filter constant 0.0010; discard Negative Intensities background; drift correction with respect to first angle; custom K0 guess angles for camera positions. The reconstructed images were further processed for maximum-intensity projections with softWoRx 6.1.1. Pixel registration was corrected to be less than 1 pixel for all channels using 100 nm Tetraspeck beads.

## Transwell assay

For the transwell migration assay, 300 μL of FBS-free medium containing 2 × 10⁴ MCF7 cells was added to the upper chamber of a transwell insert (#3422, Corning, USA), while 750 μL of DMEM supplemented with 10% FBS was placed in the lower chamber. After incubation for 24 h at 37 °C under 5% CO₂, cells were fixed with 4% paraformaldehyde for 20 min. Migrated cells on the lower surface of the membrane were stained with 0.1% crystal violet for 20 min at room temperature. Non-migrated cells on the upper surface were gently removed using a cotton swab. Images were acquired from three randomly selected fields using an Olympus FV3000RS confocal microscope.

## Live cell time-lapse imaging and trajectory analysis

HT1080 cells stably expressing mScarlet-tagged Arpin variants were seeded at low density on glass-bottom dishes coated with fibronectin and allowed to attach in complete DMEM. Cell migration was monitored using the PE Operetta High Content Screening System (PerkinElmer, USA) at 37 °C and 5% CO₂ for 6h. The instrument automatically scanned each well at 10× magnification, capturing images from 9 regions per well. It simultaneously acquired 3 channels (bright-field observation, differential phase contrast (DPC), and mScarlet). For Movie S1, the images were taken once in 10 min for 6 hours. Image analysis was performed using Harmony 4.0 software, enabling automated imaging then analysis during the screening process. The main steps of the analysis protocol involved identifying transfected cells via the red fluorescence channel and quantifying total cell counts per well using the DPC channel. To minimize positional drift caused by early plate movement, imaging was initiated from the fifth time point, when the plate was stably settled on the stage. The following criteria were obtained at all time points, including accumulated distance (μm), displacement (μm), speed (μm/s), and directional persistence (straightness). In a protocol refinement step, cells were filtered by selecting those with "Begin" as the start type and "End" as the end type prior to statistical analysis. Cells labeled as Split, Border, Merged, or Cosmos during tracking were excluded to minimize artifacts and ensure accurate quantification of individual cell trajectories. Time-lapse data were analyzed using Harmony 4.0 software (PerkinElmer, USA).

## Western Blot

MCF7 cell lysates were prepared for protein samples, separated by SDS-PAGE (40 μg per lane), followed by transfer onto PVDF membranes. After blocking with 5% non-fat milk in TBST, the membranes were incubated with primary antibodies against Arpin (Cusabio, China) and actin (Abmart, China). Subsequently, membranes were incubated with appropriate HRP-conjugated secondary antibodies: goat anti-rabbit (Cell Signaling Technology, USA) or goat anti-mouse (Abmart, China). Protein signals were detected using the Tanon 5200 Multi-imaging System (Tanon, China) and quantified with ImageJ software.

## Crosslinking

To construct the crosslinked D-aCT peptide, Arpin’s CT with an N-terminal Cys and a following spacer (-[SSGG]_4_-) was recombinantly expressed in *E. coli* (Table S2). The expressed peptide possesses additional N-terminal His-tag and SUMO-tag, enabling the purification via Ni-affinity chromatography. Subsequently, the additional tags were removed by overnight Ulp1 cleavage. The cleaved peptides were further purified by ion exchange chromatography without reducing agent. Subsequently, the purified peptides were incubated at room temperature with BM(PEG)_3_ (1,11-bis(maleimido)triethylene glycol) at a molar ratio of 1:1.3 for 30 minutes. After incubation, the crosslinked fraction was isolated from the intact fraction by SEC via a Superdex 75 column (GE Healthcare).

## AI-aided design of the dual-tailed peptide

Due to the absence of high-resolution experimental structure for the Homo sapiens Arpin-bound Arp 2/3 complex, the homologous structure of Actin obtained from Bos taurus was employed as the foundational template. The cryo-EM-derived structure, PDB entry 7JPN (resolution: 3.2 Å), was selected as the initial model, which represents the assembly state of the Arpin-actin complex. For computational design, the α-helical region (residues number 194–206, as shown in Figure S1, the canonical binding site) was selected as the region to be redesigned. For this helical segment, over 600 electron densities were produced using our proprietary generative algorithm. With iterative refinement, 2,292 structural samples were obtained through employing our in-house developed Deep-learning-based segmentation model to identify the residue type and conformation.

The CryoEM structure of the Arp2/3complex with bound NPFs (PDB entry: 6UHC, resolution: 3.9 Å) and Arpin (PDB 7JPN, resolution: 3.2 Å) was utilized as a reference when there were no human Arpin-bound complex structures at both binding sites. Potential secondary binding sites for Arpin were identified by structurally superimposing the two complexes. This aligned structure, as shown in Figure S13, served as the starting point for subsequent computational screening, with the objective of identifying designed binders with better binding affinity. Biopython-based structural analysis pipelines were used to quantify hydrogen bonds, steric conflicts, salt bridge interactions, and buried solvent-accessible surface area (SASA), and absolute binding free energies were computed using the APBS (Adaptive Poisson-Boltzmann Solver) suite.

In order to prioritize high-probability binding candidates, structural evaluations were conducted on both the canonical binding site and the putative secondary binding site, incorporating both geometric and energetic factors. Finally, six candidates were selected for further experimental validation. The details with the sequence and the geometric and energetic parameters for the suspected secondary binding as well as canonical binding show in Tables S3 and S4.

# References

1	Ben Chorin, A. *et al.* ConSurf‐DB: An accessible repository for the evolutionary conservation patterns of the majority of PDB proteins. *Protein Science* **29**, 258-267 (2020).

# Table S1. Data collection and refinement statistics.

|  | **Arpin**  **native** | **Arpin**  **derivatives (**Hg(CN)_2_**)** |
| --- | --- | --- |
| **Data collection** |  |  |
| Space group | P6_5_ | P6_5_ |
| Cell dimensions |  |  |
| a, b, c (Å) | 80.3, 80.3, 124.4 | 80.5, 80.5, 124.6 |
| α, β, γ (°) | 90, 90, 120 | 90, 90, 120 |
| Wavelength (Å) | 0.9778 | 0.9778 |
| Resolution (Å) | 46.36-1.65 (1.65-1.68) ^†^ | 40.2-2.10 (2.10-2.14) |
| *R*_merge_ | 0.125 (0.635) | 0.102 (0.226) |
| Mean I/σ(I) | 7.2 (1.8) | 17.0 (2.5) |
| Completeness (%) | 99.8 (99.8) | 99.6 (97.7) |
| Redundancy | 5.5 (5.6) | 3.6 (2.5) |
| **Refinement** |  |  |
| Resolution (Å) | 46.36-1.65 |  |
| No. of reflections | 54,508 |  |
| Reflections in test set | 2764 |  |
| *R*_work_/*R*_free_ | 0.169/0.194 |  |
| No. of atoms |  |  |
| Protein | 5798 |  |
| Water | 338 |  |
| r.m.s. deviations |  |  |
| Bond lengths (Å) | 0.0146 |  |
| Bond angles (°) | 1.2338 |  |
| Ramachandran plot |  |  |
| Favored | 348 (96.94%) |  |
| Outliers | 1 (0.28%) |  |
| Average B-factor (Å^2^) |  |  |
| All atoms | 42.0 |  |
| Water | 44.0 |  |
| Cavity water | 40.0 |  |

^†^ Values in parentheses are for the highest resolution shell.

# Table S2. Summary of dimerized peptides and proteins with corresponding SPR binding parameters

| **Native and engineered proteins and peptides** | **Sequence description** | ***K*_D_ (μM)** |
| --- | --- | --- |
| Dimeric Arpin | Full length | 14.1 |
| Monomeric Arpin | Full length | 48.9 |
| Arpin^FM^ | A double-site mutant | 63.1 |
| Arpin_CT_ | C-terminal fragment | 17.8 |
| GST-aCT | GST-GlySerSerGly-aCT | 2.5 |
| D-aCT* | Cys-(SerSerGlyGly)_4_-aCT | 5.5 |
| **Artificial dimerized dual-tailed peptides** | **Amino acid sequence** | ***K*_D_ (μM)** |
| dP_arpin_0006 | EWKTKVANKAHEA | Non-detectable |
| dP_arpin_0011 | FWKSVVASQKEQQ | Non-detectable |
| dP_arpin_0028 | AVIEQVDKASQQA | 57.8 |
| dP_arpin_0548 | DLKKIVAAIASFA | 0.70 |
| dP_arpin_1071 | AVVVVVARIVKNS | 7.1 |
| dP_arpin_1076 | DEVVAVAKVEYAA | Non-detectable |
| dP_arpin_1076 | DEVVAVAKVEYAA | Non-detectable |

* Sequence information of D-aCT for (CSSGGSSGGSSGGSSGG)GASWTDNIMAQK**S**SKGAAAEIREQGDGAEDEEWDD. To avoid the formation of unnecessary disulfide bond, the cysteine at position 13 of the CA sequence was replaced by serine, which is indicated in bold.

# Table S3. Computational screening results at the canonical binding site.

| ID | sequence | contacts | Salt bridges | clashes | binding_energy  （kcal/mol） |
| --- | --- | --- | --- | --- | --- |
| dP_arpin_0001 | QWKTKVANKAHQA | 83 | 0 | 0 | -10.679 |
| dP_arpin_0002 | EWKTKVANKAHQA | 83 | 0 | 0 | -21.9851 |
| dP_arpin_0003 | QWKTKVADKAHQA | 83 | 0 | 0 | -14.9045 |
| dP_arpin_0004 | EWKTKVADKAHQA | 83 | 0 | 0 | -24.3996 |
| dP_arpin_0005 | QWKTKVANKAHEA | 83 | 0 | 0 | -15.4532 |
| dP_arpin_0006 | EWKTKVANKAHEA | 83 | 0 | 0 | -26.3723 |
| dP_arpin_0007 | QWKTKVADKAHEA | 83 | 0 | 0 | -18.5115 |
| dP_arpin_0008 | EWKTKVADKAHEA | 83 | 0 | 0 | -27.6783 |
| dP_arpin_0009 | FWKSVVASQKQQQ | 138 | 0 | 0 | -380.142 |
| dP_arpin_0010 | FWKSVVASEKQQQ | 138 | 0 | 0 | -216.377 |
| dP_arpin_0011 | FWKSVVASQKEQQ | 138 | 0 | 0 | -375.764 |
| dP_arpin_0012 | FWKSVVASEKEQQ | 138 | 0 | 0 | -211.026 |
| dP_arpin_0013 | FWKSVVASQKQEQ | 138 | 0 | 0 | -77.7391 |
| dP_arpin_0014 | FWKSVVASEKQEQ | 138 | 0 | 0 | -196.761 |
| dP_arpin_0015 | FWKSVVASQKEEQ | 138 | 0 | 0 | -73.1259 |
| dP_arpin_0016 | FWKSVVASEKEEQ | 138 | 0 | 0 | -191.381 |
| dP_arpin_0017 | AVIQQVNKASQQA | 39 | 0 | 0 | 180.4249 |
| dP_arpin_0018 | AVIEQVNKASQQA | 39 | 0 | 0 | 168.2103 |
| dP_arpin_0019 | AVIQEVNKASEQA | 39 | 0 | 0 | 202.8576 |
| dP_arpin_0020 | AVIEEVNKASEQA | 39 | 0 | 0 | 192.5639 |
| dP_arpin_0021 | AVIQQVDKASEQA | 39 | 2 | 0 | 185.2027 |
| dP_arpin_0022 | AVIEQVDKASEQA | 39 | 2 | 0 | 173.8152 |
| dP_arpin_0023 | AVIQEVDKASEQA | 39 | 2 | 0 | 207.0622 |
| dP_arpin_0024 | AVIEEVDKASEQA | 39 | 2 | 0 | 197.3668 |
| dP_arpin_0025 | AVIQEVNKASQQA | 39 | 0 | 0 | 200.294 |
| dP_arpin_0026 | AVIEEVNKASQQA | 39 | 0 | 0 | 189.9707 |
| dP_arpin_0027 | AVIQQVDKASQQA | 39 | 2 | 0 | 182.3052 |
| dP_arpin_0028 | AVIEQVDKASQQA | 39 | 2 | 0 | 170.8352 |
| dP_arpin_0029 | AVIQEVDKASQQA | 39 | 2 | 0 | 203.8147 |
| dP_arpin_0030 | AVIEEVDKASQQA | 39 | 2 | 0 | 194.1416 |
| dP_arpin_0031 | AVIQQVNKASEQA | 39 | 0 | 0 | 182.5513 |
| dP_arpin_0032 | AVIEQVNKASEQA | 39 | 0 | 0 | 170.457 |
| dP_arpin_0033 | SLIAKVQKLVVAK | 71 | 0 | 3 | 1.535628 |
| dP_arpin_0034 | SLIAKVEKLVVAK | 71 | 2 | 3 | 0.814553 |
| dP_arpin_0535 | ATKKVATAQYQNS | 61 | 0 | 0 | 57.52925 |
| dP_arpin_0536 | ATKKVATAQYENS | 61 | 0 | 0 | 59.23949 |
| dP_arpin_0537 | ATKKVATAQYQDS | 61 | 0 | 0 | 60.77753 |
| dP_arpin_0538 | ATKKVATAQYEDS | 61 | 0 | 0 | 62.51651 |
| dP_arpin_0539 | KLVQTVTSLKTRS | 51 | 0 | 0 | 195.9413 |
| dP_arpin_0540 | KLVETVTSLKTRS | 51 | 0 | 0 | 197.7891 |
| dP_arpin_0541 | KVNHQVVKIHAAS | 55 | 0 | 0 | 459.8654 |
| dP_arpin_0542 | KVDHQVVKIHAAS | 55 | 0 | 0 | 489.6458 |
| dP_arpin_0543 | KVNHEVVKIHAAS | 55 | 0 | 0 | 468.3837 |
| dP_arpin_0544 | KVDHEVVKIHAAS | 55 | 0 | 0 | 499.7697 |
| dP_arpin_0545 | SLVVKVSAASNRA | 47 | 0 | 0 | 202.571 |
| dP_arpin_0546 | SLVVKVSAASDRA | 47 | 0 | 0 | 202.0906 |
| dP_arpin_0547 | NLKKIVAAIASFA | 50 | 0 | 0 | 163.7491 |
| dP_arpin_0548 | DLKKIVAAIASFA | 50 | 0 | 0 | 81.84044 |
| dP_arpin_0549 | DLCIKTANLAEAV | 49 | 0 | 0 | 205.747 |
| dP_arpin_0550 | KLSKTSAALAVSA | 39 | 0 | 0 | 244.2823 |
| dP_arpin_0551 | QLVASVATLSATS | 47 | 0 | 0 | 295.1066 |
| dP_arpin_0552 | ELVASVATLSATS | 47 | 0 | 0 | 295.5443 |
| dP_arpin_0553 | SLKAKVSKQKALK | 58 | 0 | 0 | 283.6236 |
| dP_arpin_0554 | SLKAKVSKEKALK | 58 | 0 | 0 | 292.9358 |
| dP_arpin_1055 | DVVRKVNQLAKSA | 41 | 0 | 0 | 88.29351 |
| dP_arpin_1056 | DVVRKVDQLAKSA | 41 | 2 | 0 | 96.35349 |
| dP_arpin_1057 | DVVRKVNELAKSA | 41 | 0 | 0 | 90.18701 |
| dP_arpin_1058 | DVVRKVDELAKSA | 41 | 2 | 0 | 99.40566 |
| dP_arpin_1059 | QIIAVVALTAVLV | 49 | 0 | 0 | 68.63249 |
| dP_arpin_1060 | EIIAVVALTAVLV | 49 | 0 | 0 | 66.52035 |
| dP_arpin_1061 | NVKALSAKKVFAS | 52 | 0 | 0 | 138.4554 |
| dP_arpin_1062 | DVKALSAKKVFAS | 52 | 0 | 0 | 184.0313 |
| dP_arpin_1063 | NVVRKVNQLAKSA | 41 | 0 | 0 | 107.4244 |
| dP_arpin_1064 | DVVRKVNQLAKSA | 41 | 0 | 0 | 88.29351 |
| dP_arpin_1065 | NVVRKVDQLAKSA | 41 | 2 | 0 | 114.7842 |
| dP_arpin_1066 | DVVRKVDQLAKSA | 41 | 2 | 0 | 96.35349 |
| dP_arpin_1067 | NVVRKVNELAKSA | 41 | 0 | 0 | 108.4894 |
| dP_arpin_1068 | DVVRKVNELAKSA | 41 | 0 | 0 | 90.18701 |
| dP_arpin_1069 | NVVRKVDELAKSA | 41 | 2 | 0 | 117.022 |
| dP_arpin_1070 | DVVRKVDELAKSA | 41 | 2 | 0 | 99.40566 |
| dP_arpin_1071 | AVVVVVARIVKNS | 37 | 0 | 0 | 190.5833 |
| dP_arpin_1072 | AVVVVVARIVKDS | 37 | 0 | 0 | 196.3851 |
| dP_arpin_1073 | DQVVAVAKVQYAA | 44 | 0 | 0 | 167.7675 |
| dP_arpin_1074 | NQVVAVAKVEYAA | 44 | 0 | 0 | 190.3265 |
| dP_arpin_1075 | DQVVAVAKVEYAA | 44 | 0 | 0 | 110.1761 |
| dP_arpin_1076 | DEVVAVAKVEYAA | 44 | 0 | 0 | 186.7769 |
| dP_arpin_1077 | AVVIVVASFIKQS | 37 | 0 | 0 | 111.8765 |
| dP_arpin_1078 | QVIQVVAVNVSAA | 41 | 0 | 0 | 139.9893 |
| dP_arpin_1079 | EVIQVVAVNVSAA | 41 | 0 | 0 | 137.2367 |
| dP_arpin_1080 | QVIEVVAVNVSAA | 41 | 0 | 0 | 142.2389 |
| dP_arpin_1081 | EVIEVVAVNVSAA | 41 | 0 | 0 | 140.2551 |
| dP_arpin_1082 | AVIIAVASIKARV | 55 | 0 | 0 | 198.9389 |

# Table S4. Computational screening results at the putative secondary binding site.

| ID | contacts | saltbridges | clashes | buried_SA | binding_energy  （kcal/mol） |
| --- | --- | --- | --- | --- | --- |
| dP_arpin_0001 | 37 | 0 | 1 | 10.35791 | 183.9926506 |
| dP_arpin_0002 | 37 | 0 | 1 | 10.35791 | 182.9153402 |
| dP_arpin_0003 | 37 | 0 | 1 | 10.35785 | 187.252262 |
| dP_arpin_0004 | 37 | 0 | 1 | 10.35785 | 186.4054686 |
| dP_arpin_0005 | 37 | 0 | 1 | 10.35492 | 199.9158263 |
| dP_arpin_0006 | 37 | 0 | 1 | 10.35492 | 199.1863452 |
| dP_arpin_0007 | 37 | 0 | 1 | 10.35498 | 204.4795064 |
| dP_arpin_0008 | 37 | 0 | 1 | 10.35498 | 203.8809939 |
| dP_arpin_0009 | 75 | 1 | 4 | 10.99414 | 90.38109819 |
| dP_arpin_0010 | 75 | 1 | 4 | 10.9903 | 104.7014706 |
| dP_arpin_0011 | 75 | 1 | 4 | 10.99414 | 87.94703737 |
| dP_arpin_0012 | 75 | 1 | 4 | 10.9903 | 102.4290464 |
| dP_arpin_0013 | 75 | 1 | 4 | 10.98547 | 120.273144 |
| dP_arpin_0014 | 75 | 1 | 4 | 10.98169 | 135.7710195 |
| dP_arpin_0015 | 75 | 1 | 4 | 10.98547 | 118.7357 |
| dP_arpin_0016 | 75 | 1 | 4 | 10.98169 | 134.1918801 |
| dP_arpin_0017 | 30 | 0 | 1 | 9.324158 | -81.64570311 |
| dP_arpin_0018 | 30 | 0 | 1 | 9.324097 | -81.14807469 |
| dP_arpin_0019 | 30 | 0 | 1 | 9.303528 | -68.92425089 |
| dP_arpin_0020 | 30 | 0 | 1 | 9.303589 | -68.27339121 |
| dP_arpin_0021 | 30 | 0 | 1 | 9.303528 | -67.0738553 |
| dP_arpin_0022 | 30 | 0 | 1 | 9.303528 | -66.49799157 |
| dP_arpin_0023 | 30 | 0 | 1 | 9.303589 | -64.94197463 |
| dP_arpin_0024 | 30 | 0 | 1 | 9.303589 | -64.38404841 |
| dP_arpin_0025 | 30 | 0 | 1 | 9.324097 | -80.14459939 |
| dP_arpin_0026 | 30 | 0 | 1 | 9.324097 | -79.52570526 |
| dP_arpin_0027 | 30 | 0 | 1 | 9.324097 | -78.10828385 |
| dP_arpin_0028 | 30 | 0 | 1 | 9.324097 | -77.55635139 |
| dP_arpin_0029 | 30 | 0 | 1 | 9.324097 | -76.41449659 |
| dP_arpin_0030 | 30 | 0 | 1 | 9.324097 | -75.80491422 |
| dP_arpin_0031 | 30 | 0 | 1 | 9.303589 | -70.9444145 |
| dP_arpin_0032 | 30 | 0 | 1 | 9.303589 | -70.33823926 |
| dP_arpin_0033 | 25 | 0 | 1 | 8.028564 | 122.5227256 |
| dP_arpin_0034 | 25 | 0 | 1 | 8.028564 | 123.2858531 |
| dP_arpin_0535 | 25 | 1 | 0 | 8.756409 | 167.7423626 |
| dP_arpin_0536 | 25 | 1 | 0 | 8.741455 | 164.1490145 |
| dP_arpin_0537 | 25 | 1 | 0 | 8.750732 | 180.6458869 |
| dP_arpin_0538 | 25 | 1 | 0 | 8.735718 | 179.2961496 |
| dP_arpin_0539 | 42 | 0 | 2 | 9.166687 | 98.03600225 |
| dP_arpin_0540 | 42 | 0 | 2 | 9.166687 | 99.16010505 |
| dP_arpin_0541 | 15 | 1 | 0 | 8.383606 | -580.0564499 |
| dP_arpin_0542 | 15 | 1 | 0 | 8.379578 | -570.0738834 |
| dP_arpin_0543 | 15 | 1 | 0 | 8.381042 | -574.5371752 |
| dP_arpin_0544 | 15 | 1 | 0 | 8.376953 | -564.4343432 |
| dP_arpin_0545 | 13 | 0 | 0 | 7.851135 | 85.84630573 |
| dP_arpin_0546 | 13 | 0 | 0 | 7.851135 | 84.48292517 |
| dP_arpin_0547 | 31 | 1 | 2 | 8.798584 | 23.67949181 |
| dP_arpin_0548 | 31 | 1 | 2 | 8.798523 | 21.27153368 |
| dP_arpin_0549 | 12 | 0 | 0 | 8.469849 | 99.03573351 |
| dP_arpin_0550 | 10 | 0 | 0 | 7.156982 | -79.79173256 |
| dP_arpin_0551 | 11 | 0 | 0 | 8.224426 | 68.87982936 |
| dP_arpin_0552 | 11 | 0 | 0 | 8.224426 | 67.72872998 |
| dP_arpin_0553 | 26 | 2 | 0 | 10.3382 | -218.2488409 |
| dP_arpin_0554 | 26 | 2 | 0 | 10.33588 | -213.8005377 |
| dP_arpin_1055 | 8 | 0 | 0 | 7.131409 | 82.17364736 |
| dP_arpin_1056 | 8 | 0 | 0 | 7.13147 | 86.35067584 |
| dP_arpin_1057 | 8 | 0 | 0 | 7.131409 | 86.44700275 |
| dP_arpin_1058 | 8 | 0 | 0 | 7.131409 | 90.95290242 |
| dP_arpin_1059 | 20 | 0 | 0 | 8.378906 | 133.9748762 |
| dP_arpin_1060 | 20 | 0 | 0 | 8.378967 | 132.1958788 |
| dP_arpin_1061 | 12 | 0 | 0 | 9.645691 | 81.19163849 |
| dP_arpin_1062 | 12 | 0 | 0 | 9.645691 | 78.96280906 |
| dP_arpin_1063 | 8 | 0 | 0 | 7.131409 | 78.80420451 |
| dP_arpin_1064 | 8 | 0 | 0 | 7.131409 | 82.17364736 |
| dP_arpin_1065 | 8 | 0 | 0 | 7.13147 | 82.94504654 |
| dP_arpin_1066 | 8 | 0 | 0 | 7.13147 | 86.35067584 |
| dP_arpin_1067 | 8 | 0 | 0 | 7.131409 | 82.87029559 |
| dP_arpin_1068 | 8 | 0 | 0 | 7.131409 | 86.44700275 |
| dP_arpin_1069 | 8 | 0 | 0 | 7.131409 | 87.37782423 |
| dP_arpin_1070 | 8 | 0 | 0 | 7.131409 | 90.95290242 |
| dP_arpin_1071 | 26 | 4 | 0 | 9.43042 | -80.00241964 |
| dP_arpin_1072 | 26 | 4 | 0 | 9.419434 | -59.73026003 |
| dP_arpin_1073 | 19 | 0 | 0 | 7.961731 | 71.94127255 |
| dP_arpin_1074 | 19 | 0 | 0 | 7.957153 | 77.40612268 |
| dP_arpin_1075 | 19 | 0 | 0 | 7.957214 | 75.86595646 |
| dP_arpin_1076 | 19 | 2 | 0 | 7.949463 | 66.15939193 |
| dP_arpin_1077 | 33 | 0 | 2 | 9.453308 | 39.88748269 |
| dP_arpin_1078 | 5 | 0 | 0 | 7.743713 | 93.99354968 |
| dP_arpin_1079 | 5 | 0 | 0 | 7.743774 | 95.00732732 |
| dP_arpin_1080 | 5 | 0 | 0 | 7.743774 | 95.45144708 |
| dP_arpin_1081 | 5 | 0 | 0 | 7.743713 | 96.4544793 |
| dP_arpin_1082 | 37 | 2 | 1 | 9.44574 | -211.3670536 |

# Figure Legends

**Figure S1. Secondary structure and sequence alignment of Arpin from different species**. Including *Danio rerio* (NM_001017780), *Xenopus tropicalis* (NM_001004898), *Bos taurus* (NM_001076304), *Mus musculus* (NM_027420), *Homo sapiens* (NM_182616). Conserved amino acid sequences crossed Arpin family are indicated by red and similar residues are indicated by yellow.

**Figure S2. (A) Cartoon representation and (B) topology diagram of Arpin promoter structure.** The six helices (α1, α2, η1, η2, η3 and η4) and β-strands (β1-β11) are highlighted in ribbon (red and yellow), and the rest residues are shown in thin lines (green).

**Figure S3. Structural superposition and comparison between Arpin and similar proteins.** (A). Structural alignment between chain A and chain B in Arpin crystal structure. Arpin was compared with (B) Rim1 (PDB entry: 6CQK) and CusF (PDB entry: 3E6Z) conformations. The r.m.s. deviations are 1.95-Å for 71 aligned Cα atoms of Rim1 (z-score 6.4), and 2.41 Å for 68 aligned Cα atoms of CusF (z-score 6.4).

**Figure S4.** **Stereo diagram of protein contact shows a close-up view of Cluster I area at the dimer interface.** Contact Cluster I lies between the β-barrels, where the N-terminus of one protomer interacts with the η4 helix of the other via hydrophobic contacts and hydrogen bonds.

**Figure S5. Opposite view of the conserved surface region on the dimer of Arpin.** 180° rotated surface view of the Arpin dimer highlights the conserved “fin keel” structure from the opposite side, confirming its bilateral conservation pattern.

**Figure S6.** **Enclosed interfacial cavity of the Arpin dimer.** The experimentally phased electron density reveals a sealed cavity situated between the two contact clusters. This cavity becomes isolated from bulk solvent upon dimer formation.

**Figure S7. Homodimerization of Arpin regulates the dynamic equilibrium of its oligomeric state and inhibitory function.** As demonstrated by (A). SEC and (B). AUC analyses in both human and zebrafish variants, full length Arpin exists in a dynamic equilibrium between monomeric and homodimeric form. (C). SEC and (D). AUC analyses of double-site mutation F95A/M97A.

**Figure S8. Sensorgram summary from SPR analysis showing interactions between Arpin variants and Arp2/3 complex.** Arp2/3 complex was immobilized on CM5 surface vs increasing concentrations of Arpin variants. All sensorgrams were globally fitted using a 1:1 binding model with Biacore Evaluation Software 3.0.

**Figure S9. Representative images of crystal violet staining after cell migration captured 24 hours after treatment.** Overexpression of Arpin and truncated peptide Arpin_CT_ and Arpin_AT_ (contain putative interaction domains with the Arp2/3 complex) induced inhibition of cell migration in MCF7 cells. However, the effect of this inhibitory binding was reduced when Arpin was converted from a homodimer to two protomers (Arpin^FM^). When Arpin^∆CT^ was overexpressed, the cell migrating ability rose sharply dramatically to a level comparable to that of cells treated only with pcDNA3.1.

**Figure S10. Structural comparison of Arpin globular core and GST homodimers.** (A). Structural measurement of the distance between C-terminal Cα atoms of the homodimer of Arpin globular core, approximately 25.0 Å, with both termini symmetrically arranged and oriented outward. (B). Structural measurement of the GST homodimer (PDB ID: 6JI6), showing an inter-terminal Cα distance of approximately 46.7 Å. The two C-termini also exhibit twofold rotational symmetry and extend outward, providing a favorable geometry for fusion.

**Figure S11. Covalent crosslinking of D-aCT peptides.** Schematic diagram of D-aCT construct, incorporating N-terminal cysteine residues and a flexible spacer for crosslinking.

**Figure S12. Binding affinity of D-aCT and AI-designed dual-tailed peptide to the Arp2/3 complex.** SPR analysis was performed with immobilized Arp2/3 complex on a CM5 sensor chip and increasing concentrations of dual-tailed peptide. 1:1 Binding curves were fitted using Biacore Evaluation Software 3.0 to determine dissociation constants.

**Figure S13. Potential secondary binding sites of Arpin were determined by structural superposition.** The CryoEM structure of the Arp2/3complex with bound NPFs (PDB 6UHC, resolution: 3.9 Å) and Arpin (PDB 7JPN, resolution: 3.2 Å) were utilized as a reference and identified by structurally superimposing the two complexes.

**Figures**

**Figure S1**

**Figure S2**

**Figure S3**

**Figure S4**

**Figure S5**

**Figure S6**

**Figure S7**

**Figure S8**

**Figure S9**

**Figure S10**

**Figure S11**

**Figure S12**

**Figure S13**

# Movie S1. Time-lapse imaging of HT1080 cell migration.

Representative 6h trajectories of single-cell migration under various Arpin construct conditions. Overexpression of full-length Arpin or engineered variants alters cell movement patterns and reduces directional persistence.
